# Gender and age differences in prevalence and incidence of child sexual abuse in Croatia

**DOI:** 10.3325/cmj.2013.54.469

**Published:** 2013-10

**Authors:** Marina Ajduković, Nika Sušac, Miroslav Rajter

**Affiliations:** Department of Social Work, Faculty of Law, University of Zagreb, Zagreb, Croatia

## Abstract

**Aim:**

To examine age and gender differences in the prevalence and incidence of child sexual abuse, the level of acquaintance of the child and the perpetrator, and correlations between experiencing family violence and sexual abuse on a nationally representative sample of 11, 13, and 16 years old children.

**Method:**

A probabilistic stratified cluster sample included 2.62% of the overall population of children aged 11 (n = 1223), 13 (n = 1188), and 16 (n = 1233) from 40 primary and 29 secondary schools. A modified version of ISPCAN Child Abuse Screening Tool – Children's Version was used. Five items referred to child sexual abuse (CSA) for all age groups.

**Results:**

In Croatia, 10.8% of children experienced some form of sexual abuse (4.8% to 16.5%, depending on the age group) during childhood and 7.7% of children experienced it during the previous year (3.7% to 11.1%, depending on the age group). Gender comparison showed no difference in the prevalence of contact sexual abuse, whereas more girls than boys experienced non-contact sexual abuse. Correlations between sexual abuse and physical and psychological abuse in the family were small, but significant.

**Conclusion:**

Comparisons with international studies show that Croatia is a country with a low prevalence of CSA. The fact that the majority of perpetrators of sexual abuse are male and female peers indicates the urgent need to address risks of sexual victimization in the health education of children.

Child sexual abuse (CSA) is defined “as the involvement of a child in sexual activity that he or she does not fully comprehend, is unable to give informed consent to, or for which the child is not developmentally prepared, or else that violates the laws or social taboos of society. Children can be sexually abused by both adults and other children who are – by virtue of their age or stage of development – in a position of responsibility, trust or power over the victim” (1). CSA has received considerable attention since the late 1970s from medical, mental health, legislative, judicial, and law enforcement professionals, as well as the media and lay public, making it the most researched form of child maltreatment. This is due to clinical and research findings that indicate considerable short and long term consequences of CSA on mental and physical health. Since the first review in the late 1980s (2), literature reviews on the consequences of child sexual maltreatment have been regularly published (3-7). A recent meta-analysis of 37 eligible longitudinal studies provided robust evidence for the association between sexual abuse and lifetime mental health difficulties (7), eg, diagnosis of anxiety disorder, depression, eating disorders, posttraumatic stress disorder, sleep disorders, and suicide attempts. Association persisted regardless of victim's gender or the age at which the abuse occurred.

Regardless of certain differences, a majority of these meta-analyses and overview studies showed that people who had been exposed to multiple-incident contact forms of CSA were more likely to develop behavioral problems and psychopathology (7,8). Some studies (9) showed that non-contact forms of abuse (eg, exposure to pornography or related sexual material, unwanted looking at genitals) produced no significant abuse-related psychological or behavioral effects.

Although a minority of studies found no associations between CSA and poor mental health in adulthood (10), a bulk of empirical data converge toward the conclusion that CSA survivors face a challenging spectrum of physical and mental health difficulties that are associated with poorer well-being and higher medical and other public services expenditures. Therefore, unbiased research of CSA prevalence is necessary for planning effective preventive and treatment strategies (11). The official statistics are not sufficient source of information, since CSA prevalence is 12.7% (127 in 1000 children) when self-report measures are used and only 0.4% (4 children in 1000) when official data are the source (12). For this purpose, well designed self-report CSA epidemiological studies are necessary.

Two recent meta-analyses on the prevalence of sexual abuse against children have reported similar findings (11,12). Pereda et al (11) found that the prevalence of sexual victimization for boys was 7.9% and for girls 19.7%, with significant difference between boys and girls (2.5 girls to one boy). Also, significant differences between different parts of the world were identified, with the lowest prevalence in Europe (9.2%) and Asia (10.1%), followed by America (15.8%) and Oceania (23.9%), and the highest prevalence in Africa (34.4%).

Stoltenborgh et al (12) found that the overall prevalence of CSA was 12% and that it was significantly higher among girls (18%) than boys (7.6%). Both meta-analyses show that methodological differences drastically affect the results. Major methodological difficulties (13) arise from differences in the definition of sexual abuse, age and gender differences in the samples, and differences in the method of data collection, eg, differences in the number and generality of the questions.

Since no epidemiological studies of sexual abuse of children have been conducted, and given the importance of these studies for planning prevention and monitoring the effectiveness of measures aimed at preventing child sexual abuse, the aim of this study is to present the epidemiological indicators of prevalence of CSA in Croatia. In accordance with this aim, the following problems and hypotheses were defined:

1. To explore age and gender differences in the childhood prevalence of CSA and incidence over the previous year. It was expected that the incidence of CSA was higher for older children. It was also expected that both the prevalence and incidence of CSA were greater among girls than boys.

2. To analyze the association between experienced family violence and CSA. It was expected that there was no correlation between the frequency of experiencing family violence and CSA.

3. To explore the level of acquaintance of the child and the perpetrator of CSA for both girls and boys. It was expected that, regardless of the victim's gender, the perpetrators would be mostly persons known to the child.

## Methods

The data were collected from February to May 2011 as a part of an international project “Balkan Epidemiological Study of Child Abuse and Neglect” (BECAN). The study was conducted in nine countries (Albania, Bosnia and Herzegovina, Bulgaria, Croatia, Greece, FYR Macedonia, Romania, Serbia, and Turkey) on nationally representative samples, with an aim of assessing victimization in the general population of children aged 11, 13, and 16. The study consists of two research components: 1) an epidemiological survey of the incidence and prevalence of physical and psychological family violence experienced by children and sexual abuse against children, both explored from the perspective of children and their parents, and 2) the analysis of recorded cases of violence against children who are in treatment by child protective services. More information on the project is available on the BECAN web page (*http://www.becan.eu/).* In this article, the focus was on Croatian national CSA data.

### Participants

A nationally representative probabilistic stratified cluster sample of pupils in Croatia was used, comprising 2.62% of the overall population of children aged 11, 13, and 16 years. It was a two-stage sample of each of the defined age populations, where for every age group the school was selected in the first stage, and whole class divisions were selected in the second stage using a proportional per size (PPS) random selection method. PPS is a group of random selection methods that control the selection of clusters in stage-wise sampling based on the information about the number of final sampling units, in this case pupils. In this study, the cumulative size method was used. In the last stage of sampling, a cluster sample was formed in which all members of the selected class divisions were included in the sample. The study included 40 primary schools (76 fifth-grade and 77 seventh-grade class divisions) and 29 secondary schools (58-second-grade class divisions) in all counties of the Republic of Croatia. At the beginning of the study, the researchers contacted 5007 pupils (1744 fifth-grade pupils and 1771 seventh-grade pupils from primary schools, and 1492-second-grade pupils from secondary schools). For various reasons (eg, lack of parental consent, own refusal, or absence from school) not all children attending randomly selected classes were included in the study. The final sample included 3644 children (1223 fifth-grade grade pupils and 1188 seventh-grade grade pupils and 1233-second-grade grade pupils). Possible bias in the resulting data due to sample attrition was corrected by non-response weighting and numerically adjusting the sample according to cohort sizes in each school. The effective sample size after weighting is shown in Table 1.

**Table 1:** Effective sample size after weighting, with respect to age and gender of participants

Age (years)	Boys n (%)	Girls n (%)	Total n
11	506 (49.0)	527 (51.0)	1033
13	496 (48.7)	522 (51.3)	1018
16	550 (48.9)	574 (51.1)	1124
Total	1552 (48.9)	1623 (51.1)	3175

### Data collection procedure

Approvals for conducting the study were issued by the ethics committee of the Faculty of Humanities and Social Sciences and the Ministry of Science, Education and Sports. According to the Croatian Ethical Code for Research with Children (14), parents of all children whose classes were sampled were informed about the study by field researchers during regular parent meetings. For all age groups of children, parents who were not able to attend the meetings were sent written information about the research. For children younger than 14 years, a written parental consent for participation was requested. For children older than 14 years, parental consent was not necessary and the decision to participate in the study was left only to the children.

Primary school children who had parental consent and all secondary school pupils were explained the purpose of the research and the process of implementation. Participants were guaranteed anonymity and confidentiality. Exceptions were explained, including the obligation of researchers to report any information on abuse obtained in direct communication with the child. Children filled out questionnaires during one school period (45 minutes) with two researchers present in the classroom. Children placed the completed questionnaires in a large envelope to ensure anonymity. At the end of the session researchers distributed thank-you notes to children, including a help-line number, which they could call if they felt upset by the contents of the questionnaire.

### Instruments

An international instrument, developed by the International Society for Prevention of Child Abuse and Neglect (ISPCAN) and UNICEF, for the assessment of the incidence and prevalence of children's exposure to abuse and neglect was used in this study. The original questionnaire ISPCAN Child Abuse Screening Tool Children's Version (ICAST-C) (15) was carefully modified for this study and details of these modifications are presented in the final international report (16). A longer version of the questionnaire, designed for students aged 13 and 16 years, consisted of 52 questions concerning the child's experiences with physical, psychological, and sexual violence. The shorter version for children aged 11 years comprised 47 questions. The questionnaire was shortened for younger children because the pilot study had shown that in most of the countries it had taken longer than one school period for children of this age to complete the questionnaire (16).

The Croatian version was validated through the implementation of two pre-testing stages. The first stage included focus groups, which were conducted with children of appropriate age (7 focus groups) and parents who had children of that age (3 focus groups). The aim was to check the comprehensiveness, clarity, and understanding, as well as the cultural appropriateness of the questions. Respondents’ interest and attentiveness to the questionnaire was also discussed. The results of these focus groups are presented in detail in a separate article (17). The second stage was the pilot study conducted in 6 grades of primary and secondary schools with children of appropriate age and the aim was to validate the procedure of gaining parental consent for the children’s participation and the entire implementation of the study. In this stage, possible problems and questions raised by children were identified so that they could be dealt with in a same way in all participating countries.

The questionnaire consists of two parts. In the first part, children report the frequency of behaviors that refer to physical and psychological violence perpetrated by family members. In the second part, which is the focus of this article, children report experienced sexual abuse, where both family members and other individuals can be specified as perpetrators. This part contains 5 items for all age groups. The contents of these items are in line with the most common definition of CSA (1) (Table 2). The term CSA has been used in psychological and medical literature describing virtually all sexual interactions between children or adolescents and significantly older persons, as well as between same-age children or adolescents when coercion is involved (10). As well, in more recent literature the term sexual victimization is often used (15,18). In this article both terms, child abuse and neglect (CAN) and sexual victimization, are used referring to experiences tapped with specific questions used in this study.

**Table 2:** Lifetime prevalence and previous year incidence of sexual abuse

	Age	n	Never experienced (No, %)	Experienced, but not in the last year (No, %)	Incidence in the last year (No, %)	Lifetime prevalence (including last year) (No, %)	Do not want to answer (No, %)	Missing (No, %)
Non-contact sexual abuse								
Made you upset by speaking to you in a sexual way or writing sexual things about you	11	1033	984 (95.2)	6 (0.6)	15 (1.5)	21 (2.1)	15 (1.4)	12 (1.2)
	13	1018	934 (91.7)	13 (1.2)	46 (4.4)	59 (5.6)	22 (2.1)	6 (0.6)
	16	1124	970 (86.3)	38 (3.4)	79 (7.1)	117 (10.5)	27 (2.4)	9 (0.8)
	Total	3176	2888 (90.9)	57 (1.8)	141 (4.4)	198 (6.2)	64 (2.0)	28 (0.9)
Made you watch a sex video or look at sexual pictures in a magazine or on computer when you did not want to	11	1033	992 (96.0)	2 (0.2)	17 (1.6)	19 (1.8)	13 (1.3)	10 (1.0)
	13	1018	965 (94.8)	9 (0.9)	20 (2.0)	29 (2.9)	16 (1.6)	7 (0.7)
	16	1124	1084 (96.4)	7 (0.7)	15 (1.1)	22 (1.8)	12 (1.1)	7 (0.6)
	Total	3176	3040 (95.7)	18 (0.6)	51 (1.6)	69 (2.2)	42 (1.3)	24 (0.8)
Made you look at their private parts or wanted to look at yours	11	1033	1001 (96.9)	6 (0.5)	5 (0.5)	11 (1.0)	12 (1.2)	9 (0.9)
	13	1018	970 (95.3)	6 (0.6)	17 (1.7)	23 (2.3)	17 (1.7)	8 (0.8)
	16	1124	1048 (93.2)	21 (1.8)	27 (2.4)	48 (4.2)	20 (1.8)	7 (0.6)
	Total	3176	3019 (95.1)	32 (1.0)	48 (1.6)	80 (2.6)	50 (1.6)	24 (0.8)
Contact sexual abuse								
Touched your private parts in a sexual way, or made you touch theirs	11	1033	1007 (97.5)	0 (0.0)	4 (0.4)	4 (0.4)	11 (1.1)	11 (1.1)
	13	1018	958 (94.1)	7 (0.7)	28 (2.8)	35 (3.5)	18 (1.7)	7 (0.7)
	16	1124	1018 (90.6)	24 (2.1)	48 (4.3)	72 (6.4)	26 (2.3)	8 (0.7)
	Total	3176	2984 (94.0)	31 (1.0)	81 (2.5)	112 (3.5)	55 (1.7)	26 (0.8%)
Tried to have sex with you when you did not want them to	11	1033	1010 (97.8)	1 (0.1)	4 (0.3)	5 (0.4)	10 (1.0)	9 (0.9)
	13	1018	982 (96.4)	7 (0.7)	9 (0.9)	16 (1.6)	15 (1.5)	5 (0.5)
	16	1124	1056 (93.9)	15 (1.4)	27 (2.4)	42 (3.8)	19 (1.7)	6 (0.5)
	Total	3176	3048 (96.0)	23 (0.7)	39 (1.2)	62 (1.9)	45 (1.4)	20 (0.6)

For examining psychological aggression 9 items were used (eg, “Has anyone in your family and living in your home refused to speak to you (ignored you)?”) and 9 were also used for psychological abuse (eg, “Has anyone in your family and living in your home said that they wished you were dead or had never been born?”). Physical violence was also measured, using 6 items for physical punishment (“Has anyone from your family done something such as spanked you on the bottom with bare hand?”) and 11 for physical abuse (“Has anyone from your family done something such as intentionally burned or scalded you?”).

The participants’ task was to indicate on all items whether they had experienced certain behavior and, if so, how often that had happened in the previous year. Answers regarding the frequency of these experiences in the previous year (last 12 months) included: “Once or twice a year (1-2 times),” “Several times a year (3-5 times),” “Monthly or bimonthly (6-12 times),” “Several times a month (13-50 times),” and “Once a week or more often (more than 50 times).” Along with these, following answers were also offered: “Not in the last year, but it has happened to me before,” “Never in my life,” and “I don't want to answer.” Such range of responses allows assessment of both the prevalence and one year incidence of CAN. The term “prevalence” is used for the proportion of children in the sample who experienced some form of CSA during childhood including the previous year. The term “incidence” refers to the proportion of children in the sample who experienced some form of CSA during the previous year (year 2010), regardless of whether they had experienced it before or not.

Participants who reported having experienced some sexually violent behavior in the previous year or earlier in life were asked to specify all the people who had behaved in such a way. First, they were supposed to mark whether this person was an “adult man,” “adult woman,” “child/adolescent male,” or “child/adolescent female,” and then if he/she was a stranger, a person they knew, or someone who was related to them. For questions regarding the experience of family violence, the list of possible perpetrators included parents, siblings, and other relatives who lived in the same household.

In our study, the coefficient of internal reliability of the scale (Cronbach α) for the 5-items scale of sexual victimization was 0.68. The validation of the ICAST-C, conducted in 4 countries with a convenience sample of 571 children aged from 12 to 17 (15), found a slightly higher coefficient of internal consistency (0.72).

### Statistical analyses

The results are presented for the entire sample and each age group. When the prevalence and incidence of experienced abuse were analyzed, answers were dichotomized as “Yes” and “No.” Reporting any form of sexual victimization was considered a “Yes” answer, that is, if the child indicated having experienced any sexually abusive behavior within the past 12 months (regardless of its frequency in that previous year), the incidence was coded as a “Yes” and if the child indicated such an experience in the past 12 months or before (“Not in the last year, but it has happened to me before”), the prevalence was coded as a “Yes” answer. Age and gender differences were tested using χ^2^ test.

Results were analyzed separately for 1) non-contact sexual abuse, which is used as the more lenient criterion for determining sexual victimization and 2) sexual abuse involving direct sexual contact, which is a more severe form of victimization. Such division of sexual abuse of children into non-contact and contact violence is common (9,11,12,19). It should be noted that this does not mean that these two categories are mutually exclusive, that is, some children might have had experiences within both categories. Data about the frequency of experienced victimization during the previous year were coded using a scale from 1 to 5 (using the answers described in the section on the instruments that were used) and correlations between types of victimization were calculated using the Pearson correlation coefficient. Although the use of non-parametric statistical tests would be more appropriate because of the asymmetrical form of distributions, parametric tests were used because when weighting participants’ results, it is not possible to conclude on the ranks of these results due to fractionation of the number of participants. Cramer’s V was used as an indicator of effect sizes for χ^2^ tests.

The percentages related to perpetrators of CSA were calculated for each form of victimization for each perpetrator marked in this category of abuse. Each child could choose more than one of 12 possible perpetrators, which limited the availability of statistical analyses that could be used, such as χ^2^ test. Therefore, in these comparisons descriptive analyses and descriptions of trends were used. The level of statistical significance was set at *P* < 0.05. Statistical analyses were performed with the statistical package SPSS 18 (20).

## Results

The prevalence of each of sexually victimizing behaviors increased with age, except being forced to watch photographs/videos of sexual content, which was most present in 13-year-olds (Table 2).

There were significant age-related differences in exposure to sexual abuse in general, both non-contact and contact type, but it should be noted that Cramer’s V showed that the effect sizes of these age differences were weak, since they were all below 0.20 (Table 3). As expected, the number of children who experienced sexually violent acts increases with age. In each age group, the proportion of children who experienced non-contact sexual abuse was higher than of those who experienced contact sexual abuse.

**Table 3:** Age differences in lifetime prevalence and previous year incidence of child sexual abuse (CSA)

	Category	Whole sample (No, %)	11 y old (No, %)	13 y old (No, %)	16 y old (No, %)	χ^2^	*P*	Cramer's V
Lifetime	Non-contact CSA	268 (8.8)	46 (4.6)	83 (8.5)	139 (12.9)	44.538	≤0.001	0.121
	Contact CSA	145 (4.7)	7 (0.7)	46 (4.6)	92 (8.5)	70.825	≤0.001	0.152
	Total CSA	331 (10.8)	48 (4.8)	105 (10.7)	178 (16.5)	74.357	≤0.001	0.156
Previous year	Non-contact CSA	191 (6.3)	34 (3.4)	64 (6.5)	93 (8.7)	24.452	≤0.001	0.090
	Contact CSA	106 (3.4)	7 (0.7)	34 (3.4)	65 (6.0)	44.302	≤0.001	0.120
	Total CSA	236 (7.7)	37 (3.7)	80 (8.1)	119 (11.1)	40.250	≤0.001	0.115

Of the total number of those who experienced some form of sexually abusive behavior in their lifetime, 209 (6.6%) experienced one form, 85 (2.7%) two forms, and 42 (1.3%) three or more different forms of sexual abuse.

Correlation between the prevalence of experienced contact and non-contact sexual abuse was 0.404 (*P* ≤ 0.001). There were some differences between correlations for each age group, but all were significant at *P* < 0.001. For 11-year-olds, this correlation was 0.264, for 13-year-olds 0.387, and for 16-year-olds 0.434. When incidence in the previous year is considered, correlation between contact and non-contact sexual abuse was 0.436 (*P* ≤ 0.001) for the whole sample, and for each age group it was 0.229 (*P* ≤ 0.001), 0.399 (*P* ≤ 0.001), and 0.502 (*P* ≤ 0.001), respectively.

In the youngest age group, an approximately equal number of boys and girls experienced some form of sexual victimization in their lives. On the other hand, in the older age groups more girls than boys were subjected to these behaviors during childhood, but only when it came to non-contact sexual abuse. In the case of contact sexual abuse, there were no significant gender differences in any of the age groups (Table 4).

**Table 4:** Lifetime prevalence of child sexual abuse (CSA) by gender

Category	Age (years)	Boys (No, %)	Girls (No, %)	χ^2^	*P*	Cramer's V
Non-contact CSA	11	25 (5.1)	22 (4.3)	0.373	0.542	-
	13	27 (5.8)	56 (11.0)	8.591	0.003	0.094
	16	47 (8.9)	92 (16.8)	15.140	≤0.001	0.119
	Total	98 (6.6)	170 (10.8)	17.188	≤0.001	0.075
Contact CSA	11	3 (0.6)	4 (0.8)	0.105	0.746	-
	13	18 (3.8)	28 (5.5)	1.618	0.203	-
	16	40 (7.5)	52 (9.4)	1.220	0.269	-
	Total	61 (4.1)	84 (5.3)	2.719	0.099	-
Total CSA	11	26 (5.3)	22 (4.3)	0.548	0.459	-
	13	36 (7.6)	70 (13.7)	9.540	0.002	0.098
	16	66 (12.5)	112 (20.4)	12.178	≤0.001	0.106
	Total	128 (8.6)	204 (13.0)	15.375	≤0.001	0.071

When data for the previous year are considered, more girls than boys experienced some form of non-contact sexual victimization only in 13 years old group. As for prevalence, there were also no gender differences in any of the age groups regarding the incidence of contact sexual abuse. All gender differences that were significant, both for prevalence and the previous year victimization, had very low effect sizes (Tables 4 and 5).

**Table 5:** Previous year incidence of child sexual abuse (CSA) by gender

Category	Age (years)	Boys (No, %)	Girls (No, %)	χ^2^	*P*	Cramer's V
Non-contact CSA	11	19 (3.9)	15 (2.9)	0.697	0.404	-
	13	20 (4.3)	44 (8.6)	7.657	0.006	0.088
	16	38 (7.2)	55 (10.1)	2.871	0.090	-
	Total	76 (5.1)	114 (7.3)	6.098	0.014	0.045
Contact CSA	11	3 (0.6)	3 (0.6)	0.002	0.961	-
	13	13 (2.7)	21 (4.1)	1.409	0.235	-
	16	34 (6.4)	32 (5.8)	0.180	0.671	-
	Total	50 (3.3)	56 (3.5)	0.112	0.738	-
Total CSA	11	21 (4.3)	15 (2.9)	1.329	0.249	-
	13	25 (5.3)	55 (10.8)	9.984	0.002	0.101
	16	54 (10.2)	66 (12.1)	0.916	0.339	-
	Total	100 (6.7)	136 (8.7)	4.173	0.041	0.037

### Violence against children in the family and CSA

Although correlations between prevalence and incidence of experiencing different forms of violence in the family and prevalence and incidence of CSA were significant, they were extremely low (from 0.112 to 0.217 for prevalence and 0.083 to 0.171 for incidence) (Table 6). Experiencing sexual abuse was practically unrelated to experiencing physical and psychological violence in the family. Correlation analyses performed separately for each age group showed the same.

**Table 6:** Correlations between prevalence and incidence of experiencing different forms of violence in the family and prevalence and incidence of CSA (n = 2898-2975)

	Category	Psychological aggression r*	Psychological abuse r*	Physical punishment r*	Physical abuse r*
**Prevalence**	**Non-contact CSA**	0.153	0.217	0.133	0.188
	**Contact CSA**	0.112	0.204	0.116	0.141
**Incidence**	**Non-contact CSA**	0.145	0.165	0.120	0.146
	**Contact CSA**	0.099	0.171	0.083	0.107

*All *P* values ≤0.001.

Nevertheless, when the experience of CSA and experience of exposure to other forms of abuse were dichotomized, χ^2^-test showed that among the participants who experienced CSA there were also significantly more children who experienced other forms of abuse in the family than among participants who did not experience CSA (Table 7).

**Table 7:** Differences in experiencing family abuse between children who experienced sexual abuse and children who did not experience sexual abuse*

		Abuse in the family (No, %)	No abuse in the family (No, %)	χ^2^	Cramer's V
Prevalence^†^	Experienced sexual abuse	240 (75.5)	78 (24.5)	151.825	0.228
	No sexual abuse	1020 (39.2)	1581 (60.8)		
Incidence^‡^	Experienced sexual abuse	145 (64.7)	79 (35.3)	110.328	0.196
	No sexual abuse	807 (30.4)	1851 (69.6)		

*P ≤ 0.001.

^†^The term “prevalence” is used for the proportion of children in the sample who experienced some form of CSA in their lifetime.

^‡^The term “incidence” refers to the proportion of children in the sample who experienced some form of CSA during the previous year (year 2010), regardless of whether they had experienced it before or not.

### Level of acquaintance of the child and the perpetrator

The number of children who did not identify the perpetrator of sexual abuse was extremely small. In total, out of 76 boys who reported non-contact CSA, 6 did not identify the perpetrator and out of 114 girls only one did not identify the perpetrator. For contact CSA, out of 50 boys, only 2 did not identify the perpetrator. All 56 girls who reported it identified the perpetrator. It should be noted that some children indicated the age and gender of the perpetrator, but did not state the nature of their relationship, so the sum of percentages regarding the relationship to the perpetrator can be smaller than the percentage in the “Total” column. The type of relationship with perpetrators of sexual abuse is presented separately for girls and for boys (Tables 8 and 9).

**Table 8:** Perpetrators of sexual abuse (CSA) against girls that occurred in the previous year (only for girls who specified the perpetrators)

Category	Age	N c*	N p^†^	Adult male				Adult female				Child/adolescent male				Child/adolescent female			
				total (%)	unknown person (%)	person she knows (%)	a relative (%)	total (%)	unknown person (%)	person she knows (%)	a relative (%)	total (%)	unknown person (%)	person she knows (%)	a relative (%)	total (%)	unknown person (%)	person she knows (%)	a relative (%)
Non-contact CSA	11	14	15	39.1	26.4	12.8	0.0	3.6	3.6	0.0	0.0	51.5	9.3	19.9	18.8	5.7	0.0	5.7	0.0
	13	44	55	29.9	20.8	4.4	4.7	3.4	0.0	2.3	1.1	64.4	13.3	45.4	5.7	2.3	0.0	2.3	0.0
	16	55	74	41.9	28.5	10.2	3.2	0.0	0.0	0.0	0.0	56.9	20.3	32.8	3.8	1.2	0.0	1.2	0.0
Contact CSA	11	3	3	23.2	23.2	0.0	0.0	0.0	0.0	0.0	0.0	76.8	0.0	46.4	30.4	0.0	0.0	0.0	0.0
	13	21	21	15.6	0.0	15.6	0.0	6.1	0.0	6.1	0.0	63.2	0.0	52.8	3.7	15.1	0.0	15.1	0.0
	16	32	34	38.6	12.9	23.0	2.7	1.6	0.0	1.6	0.0	59.9	2.3	57.6	0.0	0.0	0.0	0.0	0.0

*Number of children who experienced CSA and indicated the perpetrator.

†Number of indicated perpetrators of CSA.

**Table 9:** Perpetrators of sexual abuse (CSA) against boys that occurred in the previous year (only for boys who specified the perpetrators)

Category	Age	N c*	N p^†^	Adult male				Adult female				Child/adolescent male				Child/adolescent female			
				total (%)	unknown person (%)	person he knows (%)	a relative (%)	total (%)	unknown person (%)	person he knows (%)	a relative (%)	total (%)	unknown person (%)	person he knows (%)	a relative (%)	total (%)	unknown person (%)	person he knows (%)	a relative (%)
Non-contact CSA	11	19	20	54.2	30.1	16.1	8.0	8.2	0.0	3.9	0.0	34.2	0.0	25.2	5.2	3.5	0.0	3.5	0.0
	13	20	23	22.5	3.0	10.0	5.3	0.0	0.0	0.0	0.0	33.9	4.1	23.0	4.2	43.5	0.0	43.5	0.0
	16	32	41	0.6	0.6	0.0	0.0	2.2	0.0	2.2	0.0	15.5	6.4	6.3	2.7	81.7	13.7	63.1	4.9
Contact CSA	11	3	3	0.0	0.0	0.0	0.0	0.0	0.0	0.0	0.0	0.0	0.0	0.0	0.0	100.0	0.0	73.9	0.0
	13	13	15	0.0	0.0	0.0	0.0	3.7	0.0	3.7	0.0	9.6	4.8	4.8	0.0	86.8	0.0	72.4	14.4
	16	33	37	0.0	0.0	0.0	0.0	5.0	0.0	5.0	0.0	3.0	0.0	3.0	0.0	92.0	11.0	81.0	0.0

*Number of children who experienced CSA and indicated the perpetrator.

†Number of indicated perpetrators of CSA.

Girls indicated boys (child/adolescent male) as the most common perpetrators of sexual abuse, regardless of whether it involved direct contact or not, followed by adult men. These were mostly boys they already knew, while adult men were most often unknown to them, except when it comes to contact CSA experienced by 13 and 16 years old girls, when these were mostly adult men known to them. On the other hand, younger boys indicated adult men as most frequent perpetrators of non-contact sexual abuse, but as their age increased, girls become the most frequent perpetrators. Also, when it comes to contact sexual abuse, girls (child/adolescent female) were listed as perpetrators in 87% or more cases in all age groups.

## Discussion

In Croatia, 10.8% of children between 11 and 16 years experienced some form of sexual abuse during childhood including the year preceding the study (range from 4.8% to 16.5% by age group), while 7.7% of them experienced it in the year preceding the study (range from 3.7% to 11.1% by age group). This is in line with the data obtained in the countries with the lowest incidence of CSA (15,21). In the study that used the same questionnaire with young people of a similar age (12-17 years) in four countries (India, Iceland, Russia, and Colombia) (15), the average incidence of CSA was 18%, with results for individual countries ranging from 8% in Iceland and Colombia to 34% in Russia.

The prevalence data obtained in the present study also correspond to the results of a meta-analysis of 65 studies from 22 countries (11). The CSA prevalence rate in Croatia shows that 10.8% of children experienced some form of lifetime sexual abuse, which is in the lower range of the findings of this meta-analysis for Europe. Furthermore, it is lower than indicated in the recent “ONE in FIVE” campaign, suggesting that 1 in 5 children in Europe are victims of sexual violence (22).

As for the national data, results of the present study can be compared with two other studies. Ručević (23) studied, among other types of abuse, exposure to sexually abusive behaviors of 16 years old participants. She reported that 7.3% participants had been exposed to showing of genitals and 5.0% had experienced touching of genitals. In our sample 4.2% and 6.9%, respectively, were exposed to these forms of CSA. Unlike our study, Ručević (23) used a convenience sample including young people who had been institutionalized for delinquent behavior. The prevalence of all forms of victimization in childhood, including exposure to sexual abuse, is higher for such population (24).

In a study on 18-year old graduates of secondary schools (19), which is the closest referent age sample of school population to our 16-year-olds, it was found that 13.7% of them had been subjected to sexual abuse involving direct contact, compared to 8.5% in our study. Sexual abuse in general, both contact and non-contact, was experienced by 18.1%, compared to 16.5% in our study. Lower results obtained in the present study for contact CSA may reflect an actual decline in sexual violence against children, which was recorded in some countries, such as the USA, as a result of preventive and repressive measures (25).

As expected, in this study not only CSA prevalence, but also incidence was higher for older children. The higher rate of CSA incidence for older participants (13 and 16 years compared to 11 years) in this study may reflect the developmental phase in which the interest for sexuality increases (26), putting those children at a higher risk of unwanted sexual experiences.

Regarding children’s gender, a recent meta-analysis (11) showed that the prevalence of sexual abuse for boys was lower than for girls (female to male ratio, 2.5:1). Although some gender differences in CSA between countries can be explained by differences in the research methodology, girls are consistently reporting more sexual victimization than boys (12).

Studies conducted in various countries, such as Sweden (27), Switzerland (21), USA (28), and Australia (29), have shown that girls are more exposed to CSA than boys. In our study, this was true for the 13 and 16-year old groups and only when it comes to non-contact sexual victimization. In the 11-year-old group, there was no difference in the prevalence of experienced CSA between boys and girls, which is consistent with some previous findings (30). According to the developmental hypothesis, because gender differentiation increases as children grow older, the pattern of victimization also increases, and it is less gender-specific for younger children who are more similar in their activities and physical characteristics (30).

In general, our results confirm the existence of gender differences at the expense of girls. However, the interaction analysis of age and forms of CSA shows gender differences only for non-contact sexual abuse, which represents a milder form of sexual victimization (7-9), in older age groups. This suggests that these differences should be taken into consideration in planning targeted CSA prevention programs and that these two forms of sexual abuse should be analyzed separately.

Regarding contact and non-contact CSA, previous studies showed that the overlap between these two groups of sexually abusive behaviors is quite small (31). In contrast, correlation analyses in our study showed that for prevalence these two forms of CSA shared 16.32% of common variance, and for incidence they shared 19.01% of common variance.

Regarding perpetrators of CSA, a study has shown that in the majority of cases when the victims were girls, the perpetrators were friends or acquaintances, followed by family members, and, in the smallest number of cases, strangers (32). Boys are more likely to be sexually abused by male non-family members, while girls are more likely to be abused by a male family member (33). Although our data do not fully correspond to such results, they support the conclusion that the dynamics and pattern of vulnerability to sexual abuse differ considerably for boys and girls (33).

Specifying the frequency of boys and girls as perpetrators of sexual abuse, regardless of the victim’s gender, is an important topic related to violence in youth relationships, which often has a specific gender dynamic. The problem of sexual violence and abuse in youth close relationships is a topic of numerous studies (34-37). In this perspective, it is necessary to devote additional attention to carefully planned gender-sensitive school education. It is also necessary to systematically investigate the prevalence and incidence of sexual violence and abuse in youth relationships and among the youth in general.

In the present study, correlations between sexual abuse and physical and psychological violence in the family were very low (from 0.083 to 0.217). A previous study in Croatia (38), where only adult family members (father, mother, other adult member) were possible perpetrators of child victimization, also showed no substantial correlations between sexual victimization and physical abuse. Although physical and emotional abuse and CSA tend to overlap among identified families, there were significant differences in identified risk factors and their roots (33). Nevertheless, since additional analysis showed that among the participants who experienced CSA there were also significantly more of those who experienced other forms of abuse in the family than among participants who did not experience CSA (ratio 1.9:1 for prevalence and 2.1:1 for incidence of CSA), it is important that future studies focused on the impact of victimization on children’s well-being take into consideration the effect of poly-victimization (39). It was found that poly-victimization was highly predictive for trauma symptoms and that it greatly reduced or even eliminated the association between individual type of victimization (ie, CSA) and symptomatology (39). Attention should be given to screening children who experienced different forms of violence in the family for CSA as well.

The main strength of this study is that a large representative sample of children obtained by random recruitment was used, which provided data regarding prevalence and incidence of CSA. Furthermore, the items in the questionnaire were related to specific behaviors, which allows comparison with the international studies that use same methodological approach and question sets. The incidence data for the previous year can be compared to official statistics. In general, these data can be used as a significant epidemiological “point of reference” for monitoring the prevalence and incidence of child sexual abuse in Croatia.

This study also has several limitations. The CSA data were collected using self-report questionnaires. It is possible that participants over-reported or under-reported CSA. In an extensive questionnaire, such as modified ICAST-C, there are only five questions relating to CSA, which is a relatively small number. In addition, some of the questions that might be important to assess CSA, eg, the question regarding penetration, are not included in the questionnaire. The questions were related only to the events that participants were uncomfortable with, and any sexual activity of a child younger than 14 years or a minor with an adult more than 5 years older, even if it was voluntary, was considered sexual abuse, but it remained undetected, so this also could have reduced the prevalence of CSA in this study. This questionnaire has another drawback, regarding the definition of the perpetrators. Among the possible answers related to the perpetrators, two were “child/adolescent male” and “child/adolescent female.” Such a formulation, especially for participants at the age of 11, does not enable precise conclusions on whether the perpetrators were participants’ peers or boys and girls who were 5 or more years older, which has a different legal and psychological significance.

In conclusion, although comparisons with international studies show that Croatia is among countries with low prevalence and incidence of CSA, the results call for well-planned primary preventive programs and health education of children and young people regarding the risks of sexual abuse.
